# Supplementary material for: Pretreatment with IL-15 and IL-18 rescues natural killer cells from granzyme B-mediated apoptosis after cryopreservation
Source: Nat Commun. 2024 May 10;15:3937. doi: 10.1038/s41467-024-47574-0 (PMC11087472; doi:10.1038/s41467-024-47574-0)
Supplement: Supplementary file 1 — Supplementary Information [file 41467_2024_47574_MOESM1_ESM.pdf]

SUPPLEMENTARY INFORMATION

Pretreatment With IL-15 and 18 Rescues Natural Killer Cells from Granzyme B-Mediated Apoptosis After Cryopreservation

Corresponding authors:
Abdulla Berjis*, Neil C. Sheppard*

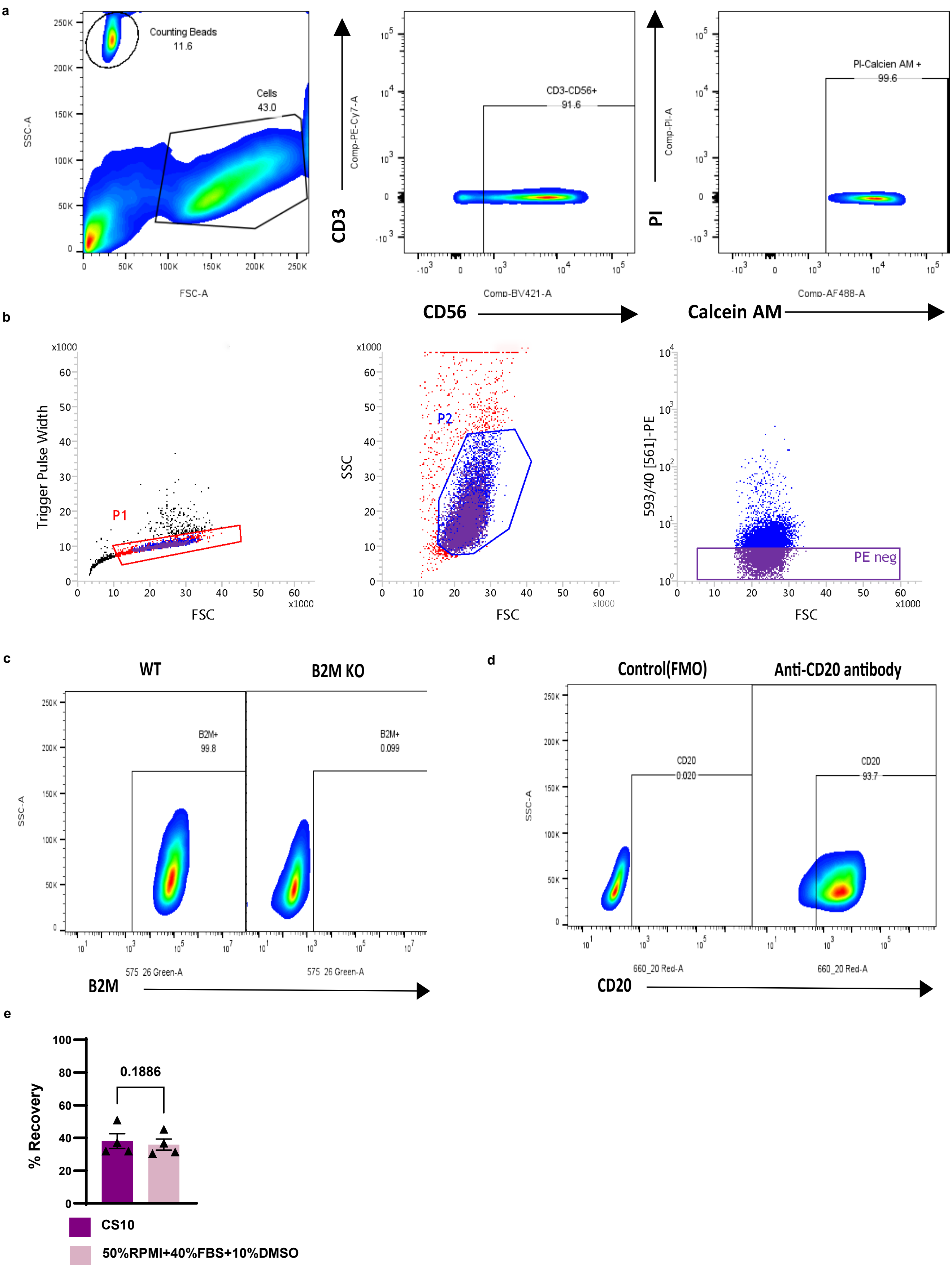

Supplementary Figure 1. Characterizing NK cell function and gene expression after Cryopreservation.

a Flow cytometry gating strategy used for all recovery and viability graphs. **b** Sorting strategy for Raji B2M KO cells. **c** Confirmation of B2M KO in Raji cells via flow cytometry. **d** CD20 expression on Jeko-1 cell line used as a target for ADCC with anti-CD20 mAb. **e** NK cell recovery after cryopreservation using published protocol (n = 4 healthy donors). Normality test was used to determine the distribution of the data, then two tailed t test was used for d. all graphs are shown as mean ± SEM.

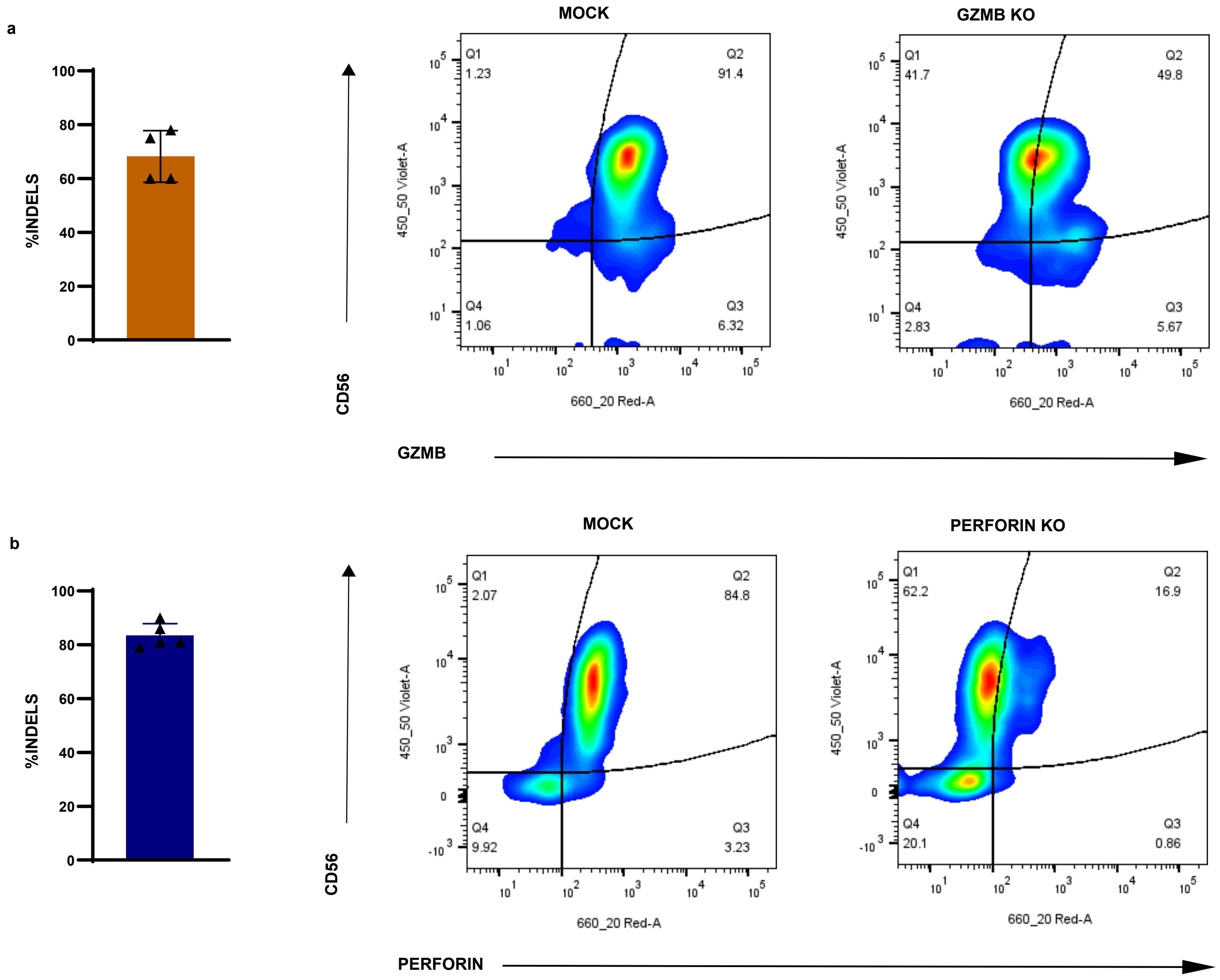

Supplementary Figure 2. Validation of GZMB and perforin KO.
a DNA %indels and flow cytometry showing GZMB KO efficiency (n = 4 healthy donors). b DNA indels and flow cytometry showing GZMB KO efficiency perforin KO NK cells efficiency (n = 5 healthy donors). All graphs are shown as mean ± SEM

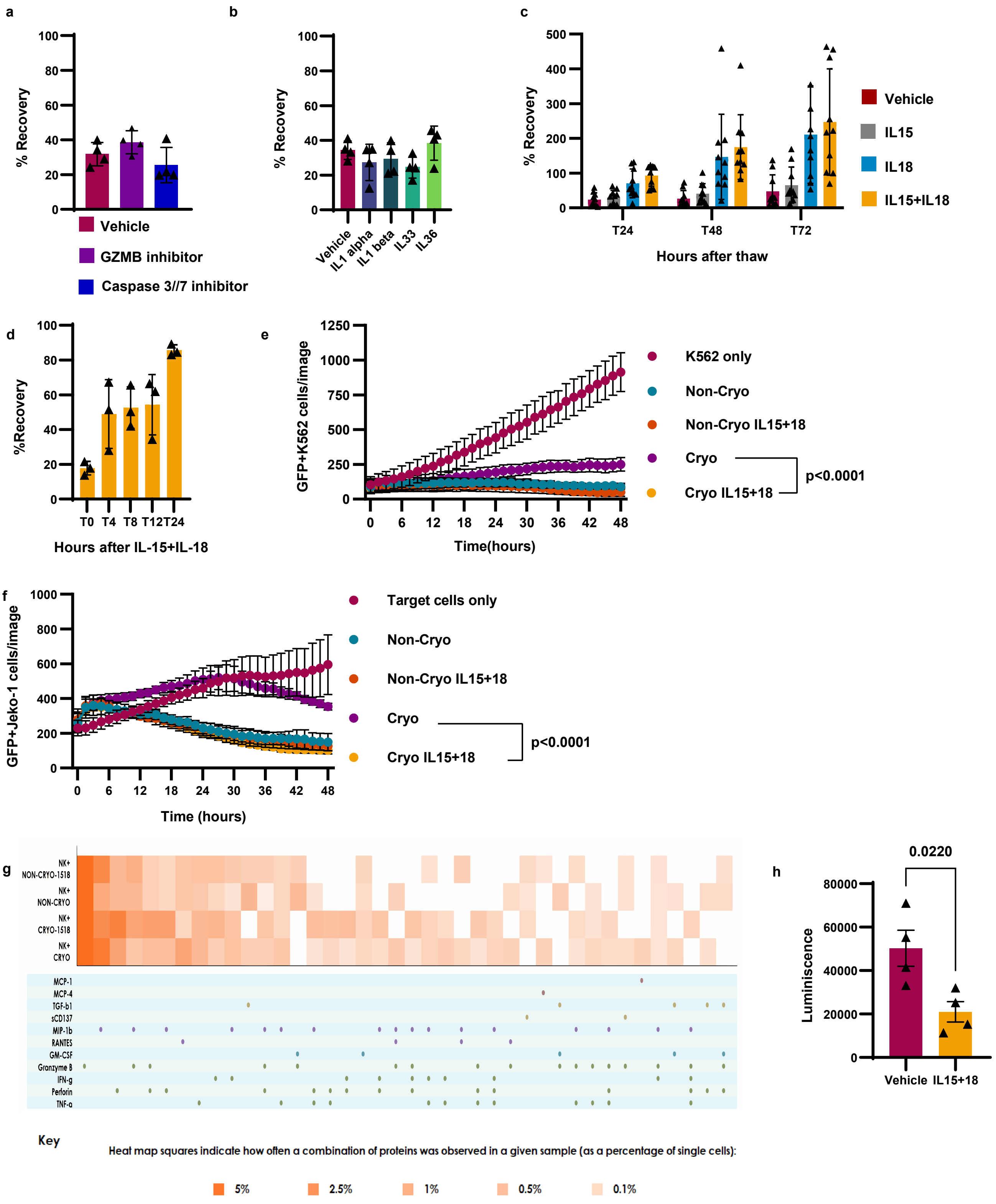

Supplementary Figure 3. The recovery and function of IL15+IL18 pretreated NK cells after cryopreservation.

a Recovery of NK cells pretreated with either a GZMB inhibitor or pan caspase inhibitor at 24 h after thawing (n = 4 healthy donors). **b** Recovery of NK cells pretreated with IL-1 cytokine family after thawing (n = 4 healthy donors). **c** NK cell recovery after pretreating NK cells with cytokines alone or in combination (n = 10 healthy donors). **d** Time course study of NK cell recovery to identify the optimal duration of pretreatment with IL-15+IL-18 (n = 3 healthy donors). **e** NK cell mediated K562 killing assay (n = 4 healthy donors). **f** NK cell mediated ADCC killing assay with anti-CD20 antibody (n = 3 healthy donors). **g** Heatmap of single cell secretome profile of IL-15+IL-18 cryopreserved, and non-cryopreserved NK cells treated with R848 for 24 h (n = 3 healthy donors).**h** Caspase 3/7 activity luminescence assay at 9 hours after thawing of vehicle and IL-15+18 treated cells. Normality test was used to determine the distribution of the data, then two tailed t test was used for h, Two-way RM ANOVA was used for e ,f and h. All graphs are shown as mean ± SEM.

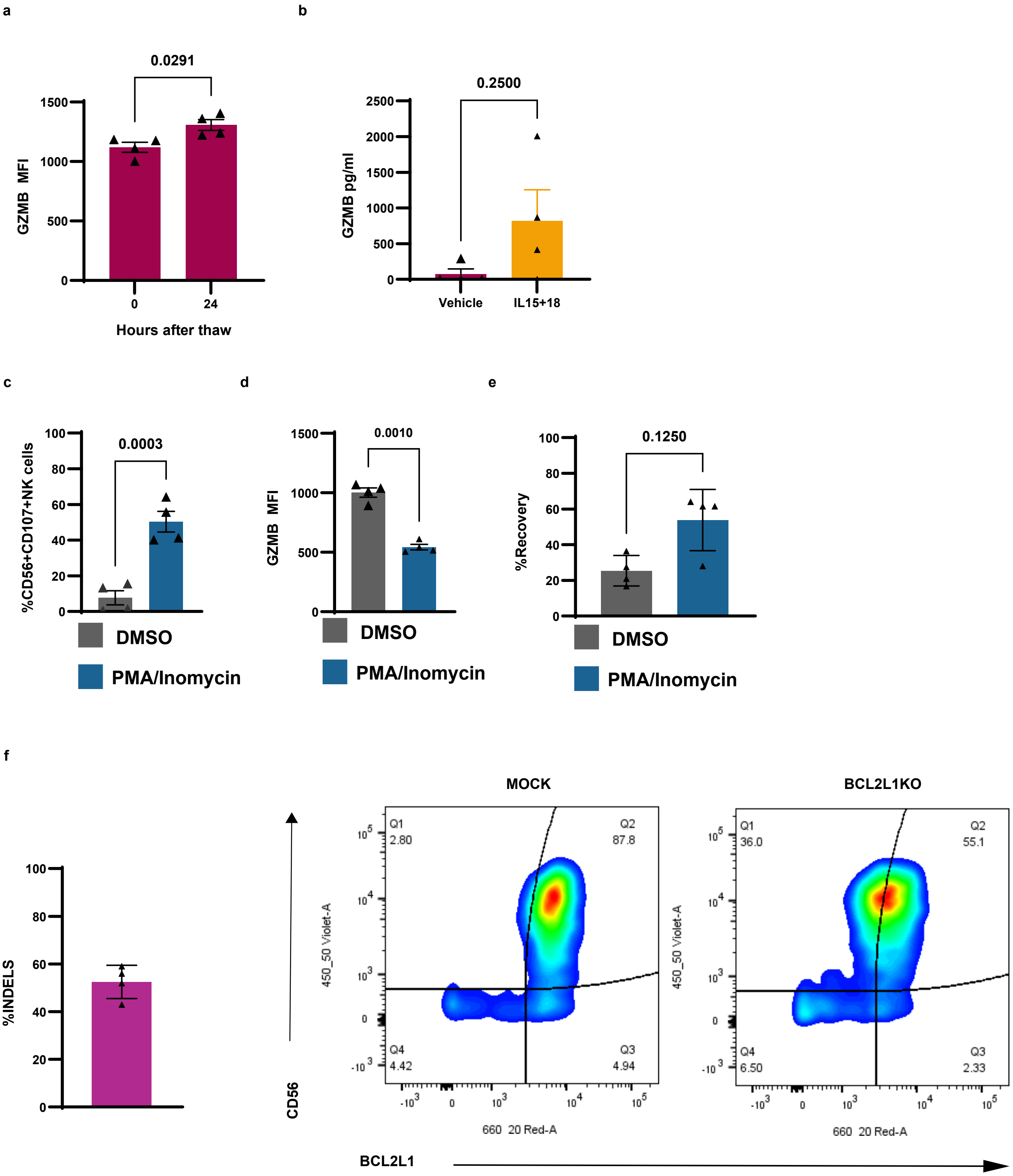

Supplementary Figure 4 GZMB levels after thawing and PMA/Ionomycin treatment

a GZMB level in IL-15+IL-18 treated cells 24 hours after thawing (n = 4 healthy donors). b GZMB secretion by IL-15+IL-18 treated cells 24 hours after thawing (n = 4 healthy donors).c DMSO or PMA/ionomycin treated NK cell degranulation after 4 hours (n = 4 healthy donors). d DMSO or PMA/ionomycin treated NK cell GZMB MFI after 4 hours (n = 4 healthy donors). e DMSO or PMA/ionomycin treated NK cell recovery after thawing (n = 4 healthy donors). f BCL2L1 KO efficiency on DNA and protein level (n = 4 healthy donors). Normality test was used to determine the distribution of the data, then two tailed t test was used for a, c, and d and non-parametric Wilcoxon matched pairs two-tailed t test was used for b and e. All graphs are shown as mean ± SEM.

a

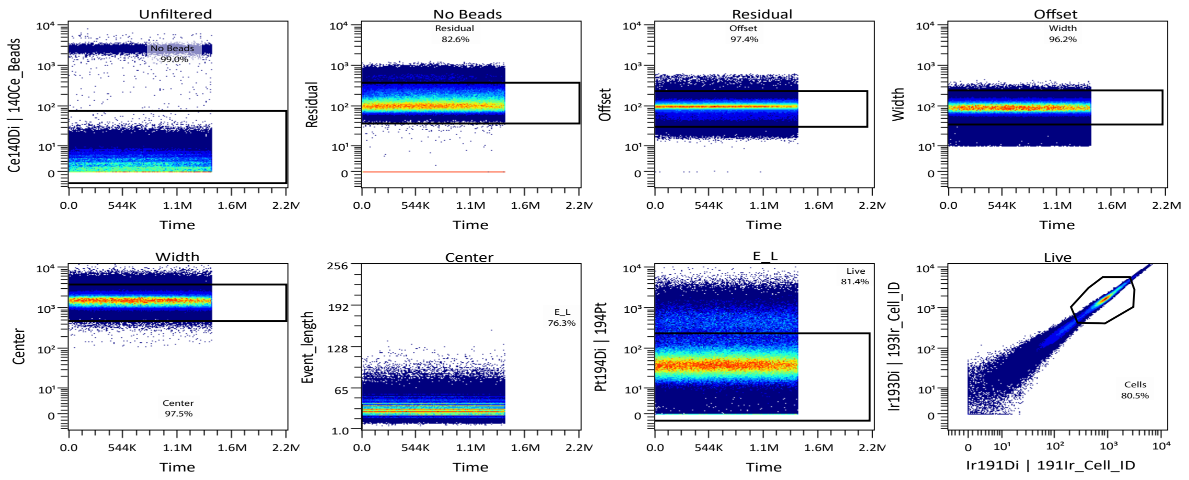

b

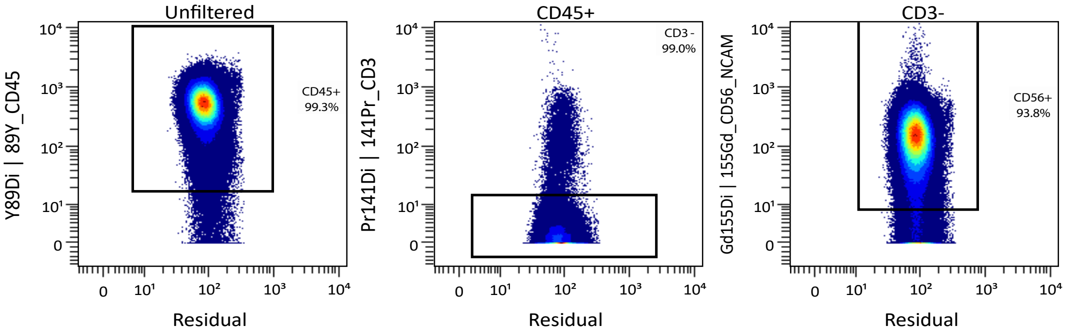

c

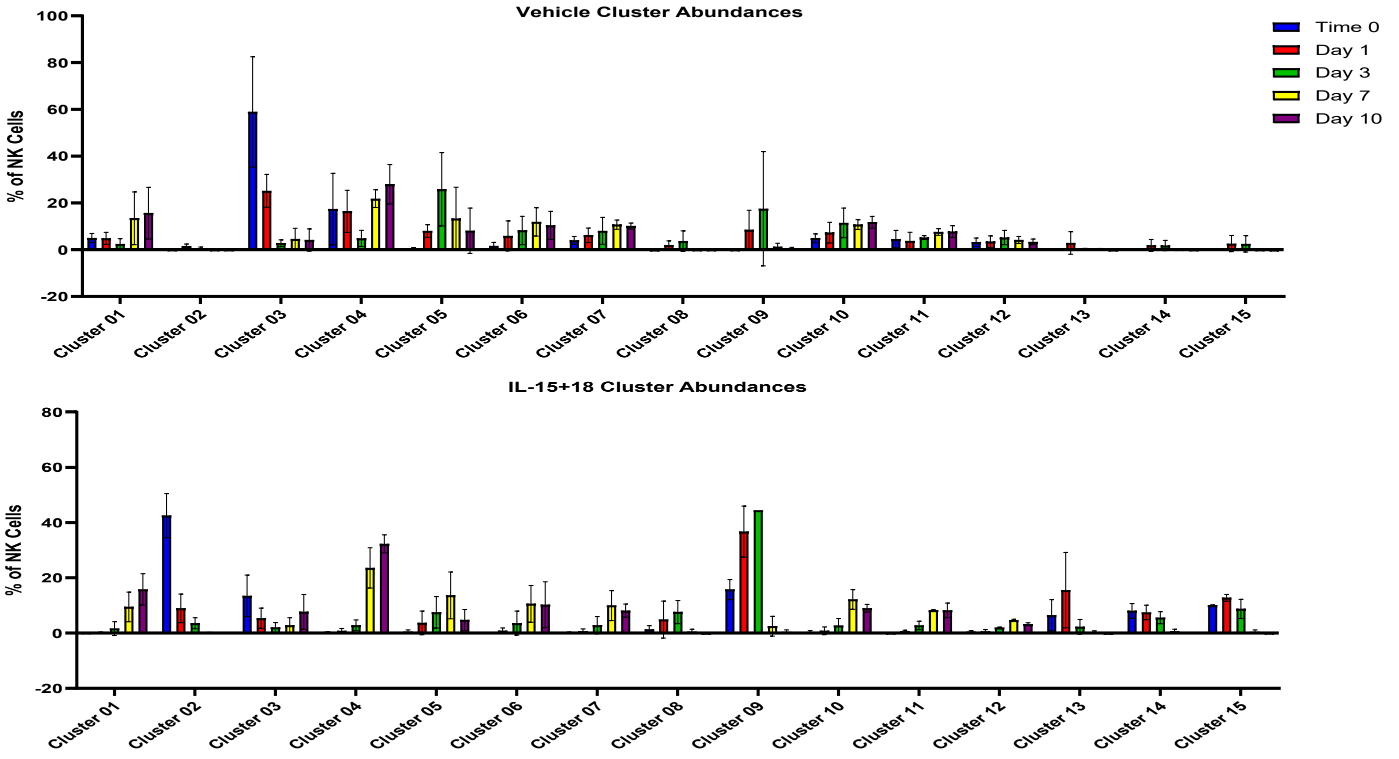

Supplementary Figure 5 NK cells phenotype after IL-15+18 treatment and Cryopreservation

a and b sample clean up and gating strategy. c NK cells percentage in each cluster at time 0 hours, 1, 3, 7, and 10 days after thawing (n = 3 healthy donors). All graphs are shown as mean ± SEM

Supplementary Tables:

Supplementary Table 1. List of antibodies and staining buffers.

Antibody	Clone	Company	Catalog
Anti-CD3 PE/Cy7	UCHT1	BioLegend	#300420
anti-CD56 BV 421	5.1H11	BioLegend	#362552
anti-CD107a Alexa Fluor 647	H4A3	BioLegend	#328612
anti-CD218a (IL-18Rα) APC	H44	BioLegend	#313814
anti-IL-15Ra (APC)	JM7A4	BioLegend	#330210
anti-Perforin (Alexa Fluor488)	dG9	BioLegend	#308108
anti-Granzyme B (Alexa Fluor 647)	GB11	BioLegend	#515405
anti-CD63 (FITC)	H5C6	BioLegend	#353006
anti-b2-microglobulin (PE)	2M2	BioLegend	#316306
anti-Bcl-xL	C.85.1	Thermo Scientific	#MA5-15142
anti-IgG (Alexa Fluor 647)	Poly4064	BioLegend	#406414
Calcein-AM	Not Applicable	BioLegend	#425201
anti-Ki-67-112Cd	Ki-67	BioLegend	CAT# 350502
anti-CD45-089Y	HI30	Standardbiotools	Cat#:3089003C
anti-CD107a-106Cd	H4A3	Standardbiotools	Cat#:3106002C
anti-CD69-113Cd	FN50	Standardbiotools	CAT#3113002C
anti-IFNg-116Cd	B27	Standardbiotools	Cat#3116002C
anti-CD3-141Pr	UCHT1	Standardbiotools	Cat#3141019C
anti-ICOS-143Nd	C398.4A	Standardbiotools	Cat#3143025C
anti-CD8a-146Nd	RPA-T8	Standardbiotools	Cat#:3146001C
anti-CD25-149Sm	2A3	Standardbiotools	Cat#:3149010C
anti-LAG-3-150Nd	11C3C65	Standardbiotools	Cat#3150030C
anti-CD2-151Eu	TS1/8	Standardbiotools	Cat#3151003C
anti-CD95 FAS-152Sm	DX2	Standardbiotools	Cat#3152017C
anti-CD62L-153Eu	DREG-56	Standardbiotools	Cat#3153004C
anti-TIGIT-154Sm	MBSA43	Standardbiotools	Cat#3154016C
anti-CD56-155Gd	B159	Standardbiotools	Cat#3155008C
anti-CD85j (LILRB1)-156Gd	GHI/75	Standardbiotools	Cat#3156020C
anti-CD137 (4-1BB)-158Gd	4B4-1	Standardbiotools	Cat#3158013C
anti-CD337 (NCR3/NKp30)-159Tb	Z25	Standardbiotools	Cat#3159017C
anti-CXCR6-160Gd	K041E5	Standardbiotools	Cat#3160016C
anti-CD161-164Dy	HP-3G10	Standardbiotools	Cat#3164009C
anti-CD314 (NKG2D)-166Er	ON72	Standardbiotools	Cat#3166016C
anti-CD366 (Tim-3)-169Tm	F38-2E2	Standardbiotools	Cat#3169028C
anti-CD226 (DNAM-1)-171Yb	DX11	Standardbiotools	Cat#3171013C
anti-CD57-176Yb	HCD57	Standardbiotools	Cat#3176019C
anti-Granzyme B-198Pt	GB11	Standardbiotools	Cat#3198002C
anti-CD16-209Bi	3G8	Standardbiotools	Cat#3209002C
anti-TNFa-114Cd	Mab11	Standardbiotools	Cat#3114002C
anti-PD1	EH12.2H7	Standardbiotools	Cat#3174020C
anti-NKB1 (KIR3DL1)-167Er	DX9	Standardbiotools	Cat#3167013C
anti-CD335-162Dy	BAB281	Standardbiotools	Cat#3162021C

anti-CD158b (KIR2DL3)-173Yb	DX27	Standardbiotools	Cat#3173010C
anti-perforin-196Pt	D48	Standardbiotools	Cat#3196002C
anti-KIR3DL1-170Er	177407	R&D systems	Cat#MAB12251
anti-KIR2DS4-163Dy	179315	R&D systems	Cat#MAB1847
anti-KIR2DL1-165Ho	HP-DM1	BioLegend	Cat# 374902
anti-NKp443 (NCR2)-144Nd	P44-8	BioLegend	Cat# 325102
anti-KIR3DL2-148Nd	539304	R&D systems	Cat# MAB2878
anti-NKG2C-147Sm	S19005E	BioLegend	Cat# 375002
anti-2B4-45N1d	2-69	BioLegend	Cat# 393502
anti-KIR2DS1-161Dy	1127B	R&D systems	Cat# MAB8887
anti-TRAIL-168Er	RIK-2	Standardbiotools	Cat# 308202
Human TruStain FcX (Fc Receptor Blocking Solution)	Not Applicable	BioLegend	# 422302
BD Cytofix/Cytoperm™	Not Applicable	BD Biosciences	# 554714
Cell-ID Cisplatin	Not Applicable	Standardbiotools	#201064
Maxpar Cell Staining Buffer	Not Applicable	Standardbiotools	# 201068
Maxpar Fix and Perm Buffer	Not Applicable	Standardbiotools	# 201067
Cell-ID Intercalator–Ir	Not Applicable	Standardbiotools	# 201192A

Supplementary Table 2. List of sequences of Guide RNAs used for the knockouts in the study.

**Gene**	**Guide RNA sequence**
β-2-Microglobulin	GAGUAGCGCGAGCACAGCUA
Granzyme B	UUUCCUUCAGGGGAGAUCAU
Perforin	GAUGCCCAGGAGGAGCAGAC
BCL2L1	GAAAGUCAACCACCAGCUCC

Supplementary Table 3. List of primer sequences used.

Gene	Primers
Granzyme B	**Forward** TATGGCAGGCTTGGTCACTC<br>**Reverse** AGGTATGCTCGCCTTCAACT
Perforin	**Forward** AGTGGAGCTGACTTTGGCC<br>**Reverse** ACCCCTCCCTAAACCTGCTA
BCL2L1	**Forward** AAAAAGGCCACAATGCGACC<br>**Reverse** TTTCGGAGAAGACGGGGGTA
