# Supplementary material for: Pretreatment with IL-15 and IL-18 rescues natural killer cells from granzyme B-mediated apoptosis after cryopreservation
Source: Nat Commun. 2024 May 10;15:3937. doi: 10.1038/s41467-024-47574-0 (PMC11087472; doi:10.1038/s41467-024-47574-0)
Supplement: Supplementary file 3 — Reporting Summary [file 41467_2024_47574_MOESM3_ESM.pdf]

nature portfolio

Corresponding author(s): Neil C. Sheppard

Last updated by author(s): Mar 29, 2024

Reporting Summary

Nature Portfolio wishes to improve the reproducibility of the work that we publish. This form provides structure for consistency and transparency in reporting. For further information on Nature Portfolio policies, see our Editorial Policies and the Editorial Policy Checklist.

Statistics

For all statistical analyses, confirm that the following items are present in the figure legend, table legend, main text, or Methods section.

n/a	Confirmed	
☐	☒	The exact sample size (*n*) for each experimental group/condition, given as a discrete number and unit of measurement
☐	☒	A statement on whether measurements were taken from distinct samples or whether the same sample was measured repeatedly
☐	☒	The statistical test(s) used AND whether they are one- or two-sided<br>*Only common tests should be described solely by name; describe more complex techniques in the Methods section.*
☐	☒	A description of all covariates tested
☐	☒	A description of any assumptions or corrections, such as tests of normality and adjustment for multiple comparisons
☐	☒	A full description of the statistical parameters including central tendency (e.g. means) or other basic estimates (e.g. regression coefficient) AND variation (e.g. standard deviation) or associated estimates of uncertainty (e.g. confidence intervals)
☐	☒	For null hypothesis testing, the test statistic (e.g. *F*, *t*, *r*) with confidence intervals, effect sizes, degrees of freedom and *P* value noted<br>*Give P values as exact values whenever suitable.*
☒	☐	For Bayesian analysis, information on the choice of priors and Markov chain Monte Carlo settings
☒	☐	For hierarchical and complex designs, identification of the appropriate level for tests and full reporting of outcomes
☒	☐	Estimates of effect sizes (e.g. Cohen's *d*, Pearson's *r*), indicating how they were calculated

Our web collection on statistics for biologists contains articles on many of the points above.

Software and code

Policy information about availability of computer code

Data collection	For Flow Cytometry the BD FACSDiva™ v8.0.1 Software was used. Incucyte® Base Analysis Software 2022B Rev3 ,Leica SP8 laser scanning confocal LAS X v3.5.7.23225. SoftMax Pro 6.2 plate reader software. All RNA sequencing data and code has been deposited Online following Nature journal instructions.
Data analysis	No software were used here beyond that stated in the Data Collection Section

For manuscripts utilizing custom algorithms or software that are central to the research but not yet described in published literature, software must be made available to editors and reviewers. We strongly encourage code deposition in a community repository (e.g. GitHub). See the Nature Portfolio guidelines for submitting code & software for further information.

Data

Policy information about availability of data

All manuscripts must include a data availability statement. This statement should provide the following information, where applicable:

- Accession codes, unique identifiers, or web links for publicly available datasets
- A description of any restrictions on data availability
- For clinical datasets or third party data, please ensure that the statement adheres to our policy

Source data are provided with this paper. The RNA sequencing data have been deposited to NCBI database under these accession codes. Figure 1 data PRJNA1071276 [https://www.ncbi.nlm.nih.gov/sra/?term=PRJNA1071276] Figure 3 data PRJNA1071169 [https://www.ncbi.nlm.nih.gov/sra/?term=PRJNA1071169].All other data will be available upon request.

Research involving human participants, their data, or biological material

Policy information about studies with human participants or human data. See also policy information about sex, gender (identity/presentation), and sexual orientation and race, ethnicity and racism.

Reporting on sex and gender	Healthy donor primary Natural Killer cells were provided by the University of Pennsylvania Human Immunology Core. Samples are deidentified for compliance with HIPAA rules, and thus only donor sex and age information is available and is provided: ND500 (Female,29) ND615(Male,24) ND578 (Male,32) ND307(Male,50) ND544(Felmae,33),ND502 (Female,56) ND584 (Male,28) ND602(Male,40) ND410(Female,59) ND618(Male,26),ND616(Male,60) ND608(Felame, 26),ND642 (Male ,28), ND569 (Female ,33)
Reporting on race, ethnicity, or other socially relevant groupings	Not available due to HIPAA
Population characteristics	Healthy adults donors between 21-65 years old from University of Pennsylvania campus which icncludes student and employee work force, comprising donors of diverse national origin, ethnicity and other socially relevant groupings.
Recruitment	Health donors were recruited via flyers and email sent University of Pennsylvania student and employee Informed consent
Ethics oversight	University of Pennsylvania Institutional review board (IRB) approved protocol (#705906).

Note that full information on the approval of the study protocol must also be provided in the manuscript.

Field-specific reporting

Please select the one below that is the best fit for your research. If you are not sure, read the appropriate sections before making your selection.

☒ Life sciences ☐ Behavioural & social sciences ☐ Ecological, evolutionary & environmental sciences

For a reference copy of the document with all sections, see nature.com/documents/nr-reporting-summary-flat.pdf

Life sciences study design

All studies must disclose on these points even when the disclosure is negative.

Sample size	We did not have any predetermined sample size for any of the experiments. When high variability was observed in the outcome in preliminary experiments as shown in figures 1b, 3b the sample size was increased to 10 healthy donors. When mid- low level variability was observed in the outcome of the preliminary experiments as shown in all other figures, the sample size was between 3-7 donors and this was based on whether we have captured enough variability to present an accurate and sold data interpretation of the biological phenomena in the assay. Based on published data and the comments from the reviewers we believe we have sufficient sample size in all experiments in latest version of the manuscript.
Data exclusions	No data was excluded for not fitting with our hypotheses.
Replication	All experiments were repeated at least 2 times. Key findings were replicated by lab members who were otherwise not involved in this project.
Randomization	Randomization was done for in vivo experiments. For in vitro experiments randomization was done when thawing and freezing different cell groups treated with cytosines or inhibitors.Experiments were tightly controlled as described in figures or method section.
Blinding	For in vivo work staff scientist injecting the mice with different groups were not aware of the of the content of the tubes. Tubes that contained different cells groups were labeled 1-5 which also included a PBS (CTRL) group. Scientist measuring tumor growth, monitoring mice was not aware of the which group or condition the mouse being measured belonged to. At the end of the in vivo experiment the blinding was lifted and mice were assigned groups or conditioned according to which treatments they received. for in vitro work blinding was not used as most are carried out by a single scientist at time therefore blinding was not possible . All data collected and analyzed were obtained form instruments that gave absolute readouts.

Reporting for specific materials, systems and methods

We require information from authors about some types of materials, experimental systems and methods used in many studies. Here, indicate whether each material, system or method listed is relevant to your study. If you are not sure if a list item applies to your research, read the appropriate section before selecting a response.

Materials & experimental systems

n/a	Involved in the study
☐	☒ Antibodies
☐	☒ Eukaryotic cell lines
☒	☐ Palaeontology and archaeology
☐	☒ Animals and other organisms
☒	☐ Clinical data
☒	☐ Dual use research of concern
☒	☐ Plants

Methods

n/a	Involved in the study
☒	☐ ChIP-seq
☐	☒ Flow cytometry
☒	☐ MRI-based neuroimaging

Antibodies

Antibodies used

Anti-CD3 PE/Cy7 (UCHT1) BioLegend #300420
anti-CD56 BV 421 (5.1H11) BioLegend #362552
anti-CD107a Alexa Fluor 647 (H4A3) BioLegend #328612
anti-CD218a (IL-18Rα) APC (H44) BioLegend #313814
anti-IL-15Ra (APC) (JM7A4) BioLegend #330210
anti-Perforin (Alexa Fluor488) (dG9) BioLegend #308108
anti-Granzyme B (Alexa Fluor 647) (GB11) BioLegend #515405
anti-CD63 (FITC) (H5C6) BioLegend #353006
anti-b2-microglobulin (PE) (2M2) BioLegend #316306
anti-Bcl-xL (C.85.1) Thermo Scientific #MA5-15142
anti-IgG (Alexa Fluor 647) (Poly4064)BioLegend #406414
anti-Ki-67-112Cd (Ki-67) BioLegend CAT# 350502
anti-CD45-089Y (HI30) Standardbiotools Cat#:3089003C
anti-CD107a-106Cd (H4A3) Standardbiotools Cat#:3106002C
anti-CD69-113Cd (FN50) Standardbiotools CAT#3113002C
anti-IFNg-116Cd (B27) Standardbiotools Cat#3116002C
anti-CD3-141Pr (UCHT1) Standardbiotools Cat#3141019C
anti-ICOS-143Nd (C398.4A) Standardbiotools Cat#3143025C
anti-CD8a-146Nd (RPA-T8) Standardbiotools Cat#:3146001C
anti-CD25-149Sm (2A3) Standardbiotools Cat#:3149010C
anti-LAG-3-150Nd (11C3C65) Standardbiotools Cat#3150030C
anti-CD2-151Eu (TS1/8) Standardbiotools Cat#3151003C
anti-CD95 FAS-152Sm (DX2) Standardbiotools Cat#3152017C
anti-CD62L-153Eu (DREG-56) Standardbiotools Cat#3153004C
anti-TIGIT-154Sm (MBSA43) Standardbiotools Cat#3154016C
anti-CD56-155Gd (B159) Standardbiotools Cat#3155008C
anti-CD85j (LILRB1)-156Gd (GHI/75) Standardbiotools Cat#3156020C
anti-CD137 (4-1BB)-158Gd (4B4-1) Standardbiotools Cat#3158013C
anti-CD337 (NCR3/NKp30)-159Tb (Z25) Standardbiotools Cat#3159017C
anti-CXCR6-160Gd (K041E5) Standardbiotools Cat#3160016C
anti-CD161-164Dy (HP-3G10) Standardbiotools Cat#3164009C
anti-CD314 (NKG2D)-166Er (ON72) Standardbiotools Cat#3166016C
anti-CD366 (Tim-3)-169Tm (F38-2E2) Standardbiotools Cat#3169028C
anti-CD226 (DNAM-1)-171Yb (DX11) Standardbiotools Cat#3171013C
anti-CD57-176Yb (HCD57) Standardbiotools Cat#3176019C
anti-Granzyme B-198Pt (GB11) Standardbiotools Cat#3198002C
anti-CD16-209Bi (3G8)Standardbiotools Cat#3209002C
anti-TNFa-114Cd (Mab11) Standardbiotools Cat#3114002C
anti-PD1 (EH12.2H7) Standardbiotools Cat#3174020C
anti-NKB1 (KIR3DL1)-167Er (DX9) Standardbiotools Cat#3167013C
anti-CD335-162Dy (BAB281) Standardbiotools Cat#3162021C
anti-CD158b (KIR2DL3)-173Yb (DX27) Standardbiotools Cat#3173010C
anti-perforin-196Pt (D48) Standardbiotools Cat#3196002C
anti-KIR3DL1-170Er (177407) R&D systems Cat#MAB12251
anti-KIR2DS4-163Dy (179315) R&D systems Cat#MAB1847
anti-KIR2DL1-165Ho (HP-DM1) BioLegend Cat# 374902

	anti-NKp443 (NCR2)-144Nd (P44-8) BioLegend Cat# 325102<br>anti-KIR3DL2-148Nd (539304) R&D systems Cat# MAB2878<br>anti-NKG2C-147Sm (S19005E) BioLegend Cat# 375002<br>anti-2B4-45N1d (2-69)BioLegend Cat# 393502<br>anti-KIR2DS1-161Dy (1127B) R&D systems Cat# MAB8887<br>anti-TRAIL-168Er (RIK-2) Standardbiotools Cat# 308202
Validation	All antibodies used in this paper were purchased form Biolegend,R&D systems,Thermo Scientific or Standard Biotools. As no antibody was made in house or received as gift from other labs all validation was done by one of the vendors above. To find the validation for any antibody used in our paper please visit these websites :<br>For Biolegend antibodies (https://www.biolegend.com/en-us),<br>For R&D systems antibodies (https://www.rndsystems.com/?gad_source=1&gclid=EAIaIQobChMIoJO0ws7thAMVYz4GAB1MxQ6sEAAYASAAEgJa4fD_BwE&gclsrc=aw.ds)<br>For Thermo Scientific antibodies (https://www.thermofisher.com/us/en/home.html)<br>For Standard Biotools antibodies (https://www.standardbio.com/)

Eukaryotic cell lines

Policy information about cell lines and Sex and Gender in Research

Cell line source(s)	Raji ,Jeko-1 and K562 cell lines from ATCC. All human cells were isolated by the Human Immunology Core at the University of Pennsylvania. Healthy donors were randomly selected and not biased towards male or female donors. Both sexes were used in our study.
Authentication	Cell lines were authenticated by ATCC through Short tandem repeat (STR) analysis.
Mycoplasma contamination	All cell lines were tested negative for Mycoplasma using Cambrex MycoAlert after culture in antibiotic-free media according to Manufacturer's instructions
Commonly misidentified lines<br>(See ICLAC register)	The website link is broken. We checked other database and Raji cell line was listed. We have sent Raji cells to ATCC and got the results confirming that the cell line sent is Raji cell line.

Animals and other research organisms

Policy information about studies involving animals; ARRIVE guidelines recommended for reporting animal research, and Sex and Gender in Research

Laboratory animals	NOD/scid/IL2rγ−/− NSG mice originally obtained from Jackson Laboratories were bred and maintained by the Stem Cell and Xenograft Core at the University of Pennsylvania in pathogen-free conditions. 6-8 weeks old male mice were used for the studies. Mice were housed in barrier mouse facility with 12 hours light-dark cycle. temperature was kept around 22±4C and humidity was kept between 45-65%.
Wild animals	No wild animals were used
Reporting on sex	Only male mice were used for the xenograft studies. Male mice were readily available as female mice were used for breeding. We do not expect that mice sex would have an impact on this aggressive xenograft model.
Field-collected samples	No field samples were collected or used for this paper.
Ethics oversight	All animal work was done under guidance of IACUC protocol at the University of Pennsylvania.

Note that full information on the approval of the study protocol must also be provided in the manuscript.

Flow Cytometry

Plots

Confirm that:

- [x] The axis labels state the marker and fluorochrome used (e.g. CD4-FITC).
- [x] The axis scales are clearly visible. Include numbers along axes only for bottom left plot of group (a 'group' is an analysis of identical markers).
- [x] All plots are contour plots with outliers or pseudocolor plots.
- [x] A numerical value for number of cells or percentage (with statistics) is provided.

Methodology

Sample preparation	Cells were stained with primary antibodies or antibodies fluorophore with in FACS buffer (2.5% FBS, PBS) and Human TruStain FcX block (BioLegend) on ice for 25 mins, washed, fixed withFixation/Permeabilization Solution Kit, With BD GolgiPlugall (BD# 554714)). All details for staining can be found in materials and methods.
Instrument	BDl LSR Fortessa (Cat#647177)
Software	BD FACSDiva™ v8.0.1 was used to collect data and Flow Jo v10.8.1 was used to analyze data .
Cell population abundance	Raji B2M KO cells were sorted multiple times until a pure B2M negative cell population was obtained.
Gating strategy	Gating strategy was showing in Supplementary Information. Gating was based on isotype controls or Fluorescence Minus One (FMO).

☒ Tick this box to confirm that a figure exemplifying the gating strategy is provided in the Supplementary Information.
